# Remote Heart Failure Symptoms Assessment After Myocardial Infarction Identifies Patients at Risk for Death

**DOI:** 10.1161/JAHA.123.032505

**Published:** 2024-01-09

**Authors:** Peter Wohlfahrt, Dominik Jenča, Vojtěch Melenovský, Josef Stehlik, John A. Spertus, Jolana Mrázková, Marek Šramko, Martin Kotrč, Michael Želízko, Věra Adámková, Jan Piťha, Josef Kautzner

**Affiliations:** ^1^ Department of Preventive Cardiology Institute for Clinical and Experimental Medicine Prague Czech Republic; ^2^ First Medical School Charles University Prague Czech Republic; ^3^ Department of Cardiology Institute for Clinical and Experimental Medicine (IKEM) Prague Czech Republic; ^4^ Third Medical School, Charles University Prague Czech Republic; ^5^ University of Utah School of Medicine Salt Lake City UT USA; ^6^ University of Missouri Kansas City’s Healthcare Institute for Innovations in Quality and Saint Luke’s Mid America Heart Institute Kansas City MO USA; ^7^ Experimental Medicine Centre Institute for Clinical and Experimental Medicine (IKEM) Prague Czech Republic; ^8^ Medical and Dentistry School Palacký University Olomouc Czech Republic

**Keywords:** heart failure, KCCQ, mortality, myocardial infarction, prognosis, symptoms, Heart Failure, Myocardial Infarction, Mortality/Survival

## Abstract

**Background:**

Heart failure is a common complication after myocardial infarction (MI) and is associated with increased mortality. Whether remote heart failure symptoms assessment after MI can improve risk stratification is unknown. The authors evaluated the association of the 23‐item Kansas City Cardiomyopathy Questionnaire (KCCQ) with all‐cause mortality after MI.

**Methods and Results:**

Prospectively collected data from consecutive patients hospitalized for MI at a large tertiary heart center between June 2017 and September 2022 were used. Patients remotely completed the KCCQ 1 month after discharge. A total of 1135 (aged 64±12 years, 26.7% women) of 1721 eligible patients completed the KCCQ. Ranges of KCCQ scores revealed that 30 (2.6%), 114 (10.0%), 274 (24.1%), and 717 (63.2%) had scores <25, 25 to 49, 50 to 74, and ≥75, respectively. During a mean follow‐up of 46 months (interquartile range, 29–61), 146 (12.9%) died. In a fully adjusted analysis, KCCQ scores <50 were independently associated with mortality (hazard ratio [HR], 6.05 for KCCQ <25, HR, 2.66 for KCCQ 25–49 versus KCCQ ≥50; both *P*<0.001). Adding the 30‐day KCCQ to clinical risk factors improved risk stratification: change in area under the curve of 2.6 (95% CI, 0.3–5.0), Brier score of −0.6 (95% CI, −1.0 to −0.2), and net reclassification improvement of 0.71 (95% CI, 0.45–1.04). KCCQ items most strongly associated with mortality were walking impairment, leg swelling, and change in symptoms.

**Conclusions:**

Remote evaluation of heart failure symptoms using the KCCQ among patients recently discharged for MI identifies patients at risk for mortality. Whether closer follow‐up and targeted therapy can reduce mortality in high‐risk patients warrants further study.

**Nonstandard Abbreviations and Acronyms**
EPHESUS: Eplerenone Post‐Acute Myocardial Infarction Heart Failure Efficacy and Survival Study
GRACE: Global Registry of Acute Coronary Events
KCCQ: Kansas City Cardiomyopathy Questionnaire
NRI: net reclassification improvement
PRO: patient‐reported outcome

**Clinical Perspective**

**What Is New?**

- Heart failure is a common complication of myocardial infarction associated with increased mortality risk.
- Whether remote evaluation of heart failure symptoms using the Kansas City Cardiomyopathy Questionnaire (KCCQ) can identify patients at increased mortality risk is unknown.
- In the present study, we show that remote evaluation of HF symptoms using the KCCQ score among patients recently discharged for myocardial infarction identifies patients at risk for mortality.

**What Are the Clinical Implications?**

- The KCCQ can be part of a toolkit for risk stratification after myocardial infarction.
- Whether closer follow‐up and targted therapy can decrease mortality risk in patients with KCCQ score <50 after myocardial infarction warrants further investigation.

Traditional, unstructured patient questioning on disease symptoms is time demanding and influenced by provider skills and subjective interpretation.^1^ Accordingly, it has been shown to be inaccurate as physicians may fail to recognize patients' functional disabilities.^2^ Patient‐reported outcomes (PROs) provide a standardized, valid, reproducible, and sensitive way to capture patient symptoms, function, and quality of life.^3^ Importantly, PROs can also predict the risk of adverse clinical events.^4^, ^5^, ^6^ In connection with modern telemedicine options, PROs may provide the opportunity to remotely identify patients who are more symptomatic and at increased risk for complications who could benefit from targeted and timely therapy.

The Kansas City Cardiomyopathy Questionnaire (KCCQ) is a heart failure (HF)–specific PRO that predicts adverse events in patients with acute^7^ and chronic HF.^4^, ^8^ After myocardial infarction (MI), KCCQ has only been used in a substudy of EPHESUS (Eplerenone Post‐Acute Myocardial Infarction Heart Failure Efficacy and Survival Study) among patients with manifest HF and reduced ejection fraction.^9^ Yet, it is unknown whether the KCCQ can be used in the general population of patients with MI to identify those at increased mortality risk.

Several prognostic models that aim to estimate the risk of all‐cause mortality, or the combined risk of all‐cause mortality or MI in patients after MI, have been developed. Among them, the Global Registry of Acute Coronary Events (GRACE) risk score has been recommended in the latest European Society of Cardiology guidelines,^10^ as it offers the best discriminative performance.^11^, ^12^ The GRACE 2.0 score uses 8 clinical variables (age, systolic blood pressure, heart rate, Killip class, creatinine, ST elevation, elevated troponin level, and cardiac arrest at admission) to predict the risk of in‐hospital, 6‐month, 1‐ and 3‐year mortality, or death or MI at 1 year.^13^ Yet, it is unknown whether the evaluation of HF symptoms has an additional predictive value to variables used in the GRACE score. HF is common after MI, developing in up to 40% of patients,^14^ and significantly increases mortality.^15^ However, HF is often diagnosed late, at a stage requiring hospital admission, which may increase mortality risk and elevate costs. We hypothesized that early HF symptoms, evaluated with the KCCQ 1 month after hospital discharge for MI, could identify patients at increased mortality risk and improve risk stratification beyond risk factors used in the GRACE score.

The aim of this study was to examine the association of the KCCQ Overall Summary score with total mortality risk in a consecutive group of patients hospitalized for MI at a large tertiary heart center.

## Methods

### Data Availability

The data that support the findings of this study are available from the corresponding author upon reasonable request.

### Population

This study used data from the prospective Institute for Clinical and Experimental Medicine Acute Myocardial Infarction Registry (AMBITION).^16^ The registry collects clinical data and biospecimens from consecutive patients hospitalized for acute coronary syndrome since June 2017 at the Institute for Clinical and Experimental Medicine, Prague, Czech Republic, a tertiary heart center with around‐the‐clock coronary intervention service. The Fourth Universal Definition of Myocardial Infarction has been used.^17^ Patients underwent a detailed interview during their hospital stay, and additional information was obtained from medical record abstraction and laboratory studies. We included consecutive patients enrolled between June 2017 and September 2022 with death ascertainment through June 2023. Data from consecutive patients hospitalized for MI were used in this analysis. Only patients with missing KCCQ score or patients who died within 1 month of hospital discharge were excluded. The study complies with the Declaration of Helsinki. The institutional review board approved the study, and all participants signed informed consent.

### Kansas City Cardiomyopathy Questionnaire

One month after discharge, patients were asked to complete the 23‐item KCCQ. Because the majority of patients did not have HF, in the questionnaire we replaced heart failure with heart disease. The patients had a choice of completing the KCCQ through an online application or on a paper form returned by regular mail. For the present analysis we used the KCCQ Overall Summary score, which we refer to as the KCCQ score. The KCCQ score ranges from 0 to 100, where higher scores indicate better function, fewer symptoms, and higher quality of life. Using published recommendations,^18^ scores were categories into ranges of very poor to poor (<25), poor to fair (25–49), fair to good (50–74), and good to excellent (≥75) health status.

### Outcomes

The primary outcome of this study was all‐cause mortality. Mortality data were provided by the Institute of Health Information and Statistics of the Czech Republic, which keeps a list of all deceased persons in the Czech Republic by law. Deaths in this study were through June 30, 2023.

### Statistical Analysis

Descriptive statistics are reported as mean±SD, median (interquartile range), or frequency (percentage). The primary outcome was all‐cause mortality, and Cox proportional hazard models were used to assess the association of KCCQ score categories with total mortality in an unadjusted model followed by adjustment for components of the validated GRACE score^13^ (age, heart rate, and systolic blood pressure at hospital admission, creatinine, maximal troponin level, double log‐transformed value, ST‐segment–elevation MI, cardiac arrest at admission and Killip class). Rather than adjusting for the GRACE score, we used adjustment for covariates used in the model. This had 2 reasons. First, the follow‐up varied in the registry, thus trimming to the prespecified time point used in the score would decrease the sample size and statistical power. Second, the GRACE score would need recalibration to our population before net reclassification improvement (NRI) could be calculated.

To account for nonlinearity, we tested restricted cubic splines adjusted for the continuous variables of age and 30‐day KCCQ scores with total mortality risk. To identify the most predictive items of the KCCQ, we used backward selection adjusted for age.

The proportional hazard assumption fulfilled the Schoenfeld residuals test. The modifying effect of age, sex, and ejection fraction on the association between KCCQ score and mortality risk was tested using interactions.

To examine the added prognostic value of KCCQ to established risk factors used from the GRACE score, we used the C index, Brier score, and NRI. The continuous NRI was calculated using the R survNRI package. All statistical tests and CIs were 2‐sided with a significance level of 0.05. Statistical analyses were conducted with R statistical software version 4.2.2 (R Foundation for Statistical Computing), SPSS version 25.0 (IBM), and STATA version 17 (StataCorp).

## Results

Between June 2017 and September 2022, 1769 patients were hospitalized for MI. Of these, 69 (3.9%) had missing KCCQ scores due to death within 1 month of hospital discharge. In total, 1135 (66.8% of eligible patients) patients completed the KCCQ 1 month after hospital discharge. Comparison of patients with available and missing KCCQ is shown in Table S1. Patients with missing KCCQ scores were slightly older and more often required cardiopulmonary resuscitation before hospital admission, while maximal troponin and mortality was similar in patients with and without KCCQ.

The mean age of the studied population was 64±12 years, with 26.7% being women, 60% having an ST‐segment–elevation MI, and 82% with Killip class I. At 30 days, 30 (2.6%) participants had a KCCQ <25, 114 (10.0%) had scores of KCCQ 25 to 49, 274 (24.1%) had scores of KCCQ 50 to 74, and 717 (63.2%) had KCCQ scores ≥75. Table 1 describes demographic and clinical characteristics by KCCQ categories.

**Table 1:** Population Demographics by KCCQ Score Categories

	KCCQ score <25 (n=30)	KCCQ score 25 to 49 (n=114)	KCCQ score 50–74 (n=274)	KCCQ score ≥75 (n=717)	Total (N=1135)	*P* for linear trend
Age, y	70.5±11.4	63.3±12.6	67.2±11.9	63.2±11.6	64.4±11.9	0.001
Female sex, n (%)	14 (46.7)	36 (31.6)	89 (32.5)	164 (22.9)	303 (26.7)	0.001
BMI, kg/m^2^	28.2±4.9	28.9±4.7	28.6±5.3	28.9±4.8	28.8±4.9	0.373
CPR, n(%)	0 (0)	4 (3.5)	8 (2.9)	22 (3.1)	34 (3.0)	0.699
Admission SBP, mm Hg	148±28	142±29	147±29	145±24	145±27	0.904
Admission DBP, mm Hg	78±17	79±14	79±14	80±13	79±13	0.407
Admission HR, beats/min	87±15	80±19	76±19	75±18	76±18	0.001
Maximal troponin, log (ng/L)	6.68±1.82	6.87±1.49	6.85±1.63	6.87±1.51	6.86±1.55	0.690
Creatinine, umol/L	108.8±75.7	106.9±105.6	100.7±69.4	87.8±35.2	93.4±57.1	0.001
HbA_1c_, mmol/mol	50.93±18.56	46.60±16.09	44.85±11.46	44.91±12.64	45.21±12.96	0.042
STEMI, n (%)	12 (40.0)	65 (57.0)	143 (52.2)	462 (64.4)	682 (60.1)	0.001
Killip class I, n (%)	14 (46.7)	81 (71.1)	217 (79.2)	619 (86.3)	931 (82.0)	0.001
EF, %	41±11	43±11	46±10	46±10	46±10	0.001
EF <40%, n (%)	12 (40.0)	36 (31.6)	65 (23.7)	141 (19.7)	254 (22.4)	0.001
Discharge medication						
ACEI/ARB, n (%)	20 (66.7)	77 (67.5)	213 (77.7)	563 (78.5)	873 (76.9)	0.011
β‐Blocker, n (%)	27 (90.0)	88 (77.2)	217 (79.2)	585 (80.8)	917 (80.8)	0.679
Statin, n (%)	24 (80.0)	105 (92.1)	264 (96.4)	690 (96.2)	1083 (95.4)	0.001
Furosemide, n (%)	20 (66.7)	37 (32.5)	74 (27.0)	120 (16.7)	251 (22.1)	0.001
Verospirone, n (%)	10 (33.3)	35 (30.7)	60 (21.9)	135 (18.8)	240 (21.1)	0.001

Data are presented as mean±SD unless otherwise indicated. ACEI indicates angiotensin‐converting enzyme inhibitor; ARB, angiotensin receptor blocker; BMI, body mass index; CPR, cardiopulmonary resuscitation; DBP, diastolic blood pressure; EF, ejection fraction; HbA_1c_, glycated hemoglobin; HR, heart rate; KCCQ, Kansas City Cardiomyopathy Questionnaire; SBP, systolic blood pressure; and STEMI, ST‐segment–elevation myocardial infarction.

### Outcome and KCCQ Predictive Value

**Figure 1:**
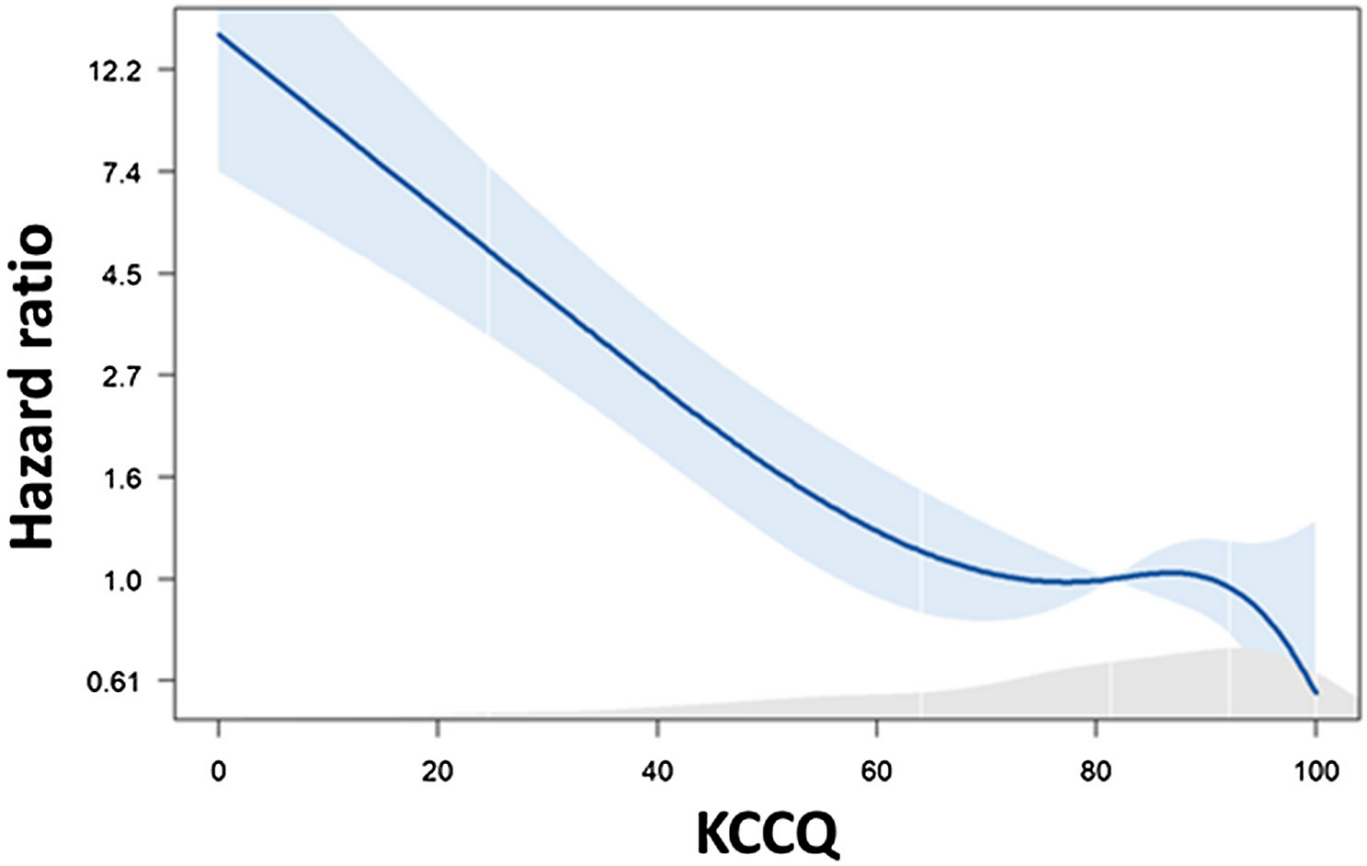
The association of Kansas City Cardiomyopathy Questionnaire (KCCQ) score with mortality risk after myocardial infarction. Data are adjusted for age. Gray shaded area represents KCCQ histogram in the population. Light blue area is the 95% CI. The reference value is the KCCQ median of 81.

**Figure 2:**
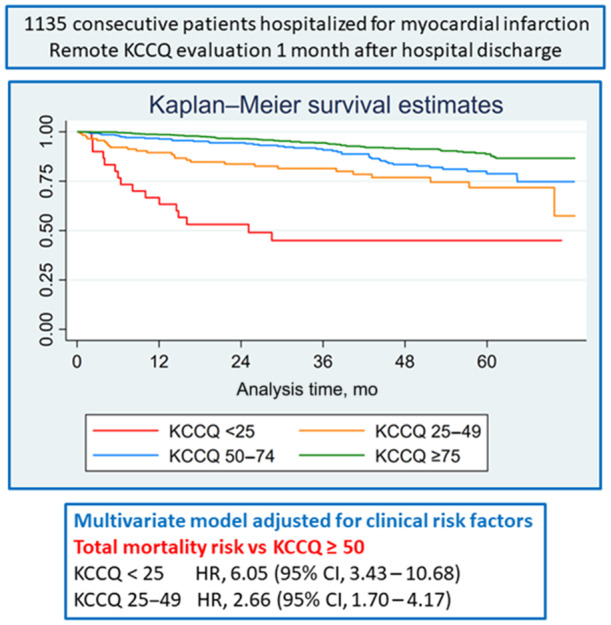
Kaplan–Meier survival curves for Kansas City Cardiomyopathy Questionnaire (KCCQ) score categories. HR indicates hazard ratio.

During a median follow‐up of 46 months (IQR, 29–61), 146 (12.9%) patients died. In the nonlinear analysis adjusted for age (Figure 1), the mortality risk increased with decreasing KCCQ score. Kaplan–Meier survival curves for KCCQ categories are shown in Figure 2. After adjusting for clinical variables included in the GRACE score, KCCQ score <50 was independently associated with mortality risk (Table 2). There were no significant interactions between the KCCQ score categories and age (*P* for interaction 0.86), sex (*P* for interaction 0.72), or systolic dysfunction with ejection fraction <40% (*P* for interaction 0.53).

**Table 2:** Risk of Mortality by KCCQ Score Categories

Model	Variable	HR (95% CI)
Unadjusted	KCCQ score	
	KCCQ <25	10.52 (6.05–18.27)
	KCCQ 25–49	3.24 (2.05–5.12)
	KCCQ 50–74	1.89 (1.28–2.80)
	KCCQ ≥75	1 (reference)
Adjusted*	KCCQ score	
	KCCQ <25	6.64 (3.67–12.01)
	KCCQ 25–49	2.78 (1.72–4.49)
	KCCQ 50–74	1.18 (0.78–1.77)
	KCCQ ≥75	1 (reference)

* Adjusted for age, sex, ejection fraction, heart rate and systolic blood pressure at hospital admission, creatinine, maximal troponin level (double log‐transformed value), ST‐segment–elevation myocardial infarction, cardiac arrest at admission, and Killip class. KCCQ indicates Kansas City Cardiomyopathy Questionnaire.

### Improvement in Discrimination, Calibration, and Stratification

In assessing mortality risk at 2 years after MI, the AUC for the 3 KCCQ score categories (KCCQ <25, 25–49, and ≥50) was 67.9 (95% CI, 61.9–73.9). The addition of 3 KCCQ categories to components of the GRACE score associated with the outcome (age, Killip class, ST‐segment–elevation MI, heart rate, creatinine level) significantly improved the C index (from AUC, 82.6 [95% CI, 78.0–87.3] to AUC, 85.3 [95% CI, 80.5–90.0]; delta AUC, 2.6 [95% CI, 0.3–5.0], *P*=0.03); and Brier score by −0.6 [95% CI, −1.0 to −0.2, *P*=0.01]). KCCQ score categories improved the continuous NRI by 0.71 (95% CI, 0.45–1.04), with a significant improvement in nonevent NRI of 0.79 (95% CI, 0.50–1.00), but without change in event NRI of −0.10 (95% CI, −0.35 to 0.18). Calibration plots for both models are shown in Figure S1. In a sensitivity analysis, the 12‐item KCCQ predictive value was similar to the 23‐item KCCQ.

### KCCQ Items and Mortality

To identify KCCQ items most strongly associated with mortality after MI, we used forward stepwise Cox regression adjusted for age. We found that responses to 3 questions were independently associated with mortality. These items included walking limitations, change in symptoms during the past 2 weeks, and leg swelling.

## Discussion

Optimally managing patients recovering from MI warrants building a longitudinal infrastructure to assess patients' risk over time so that proactive interventions can be offered to optimize patients' symptoms, function, and survival. To advance current strategies, we examined the prognostic significance of assessing patients' health status with the KCCQ 1 month after hospital discharge in a large, prospective cohort of consecutive patients recovering from an MI. We found that lower KCCQ scores, particularly <50, were independently associated with mortality risk, above and beyond clinical risk factors alone.

This study extends the field of risk stratification after hospital discharge for an MI, as we are unaware of other studies using health status measures after discharge to assess patients' long‐term prognosis. Two prior publications from the EPHESUS trial did show the KCCQ to be independently prognostic of cardiovascular death and hospitalizations, but this was in a select group of patients with diabetes or HF during their MI hospitalization.^9^, ^19^ Similarly, Dunlay et al examined the prognostic significance of the KCCQ after an admission for HF and, like the current study, found it was independently associated with survival and hospitalization.^20^ Thus, these findings of the prognostic significance of postdischarge KCCQ assessment in all‐comers with MI further supports the routine use of PROs in designing holistic, patient‐centered strategies to optimize patients' outcomes.

HF‐related quality‐of‐life impairment is common in patients after MI.^21^ In the present study, KCCQ scores <50 were present in ≈13% of patients 30 days after their MI. Interestingly, 66% of these patients were Killip class I during the hospital stay, suggesting that a large proportion of patients either developed HF symptoms after hospital discharge, or that the Killip class is insufficiently sensitive to HF symptoms. Furthermore, the KCCQ's predictive value was independent of age, sex, and left ventricular ejection fraction at hospital discharge, suggesting that the KCCQ may be a useful patient‐centered tool for identifying patients at increased mortality risk following MI.

We identified that the 3 most predictive items of the KCCQ were walking impairment, leg swelling, and change in symptoms. These symptoms are not novel and have been used by clinicians for decades; however, unstructured questioning of HF symptoms is time‐consuming, is influenced by physician subjective interpretation, and may not be consistently performed in all patients, and, as such, limits actionability. Therefore, a structured application of the KCCQ score after MI may be helpful in consistent symptom assessment and in subsequent decision‐making.

Currently, the guidelines recommend evaluation of HF symptoms during hospital stay using Killip class. The Killip classification describes lung congestion or cardiogenic shock presence, with a Killip class >I being a marker of increased risk of future events.^22^ However, evaluation of HF symptoms early after hospital discharge is not routinely or systematically performed, even though MI is a common cause of HF, with HF developing in 13% of patients 1 month after hospital discharge.^23^ A large proportion of patients with newly developed HF may therefore be missed if the infrastructure is not in place to consistently assess it, something that remote monitoring with the KCCQ can accomplish. Interestingly, the risk of death associated with HF after MI is independent of ejection fraction and greater for delayed‐ versus early‐onset HF.^15^ Thus, not identifying HF symptoms early after hospital discharge represents a missed opportunity to identify patients at increased risk in which guideline‐directed medical therapy could be proactively initiated.

Finally, we also demonstrate the feasibility of remotely assessing patients' health status, which may decrease the burden on patients and medical staff and increase the consistency of HF symptom assessment in the post‐MI period. Implementation work is needed to test that early identification of patients who develop HF symptoms after MI and are at increased risk for mortality will lead to clinical action, including additional diagnostics and targeted interventions and a subsequent favorable effect on mortality.

### Study Limitations

The primary objective of the present study was total mortality rather than cardiovascular mortality since the cause of death could not be reliably ascertained. Nevertheless, early after acute coronary syndrome, cardiovascular deaths are the major cause of mortality,^24^ and we therefore believe most deaths were related to patients' cardiovascular disease. Due to the observational nature of the analysis, we cannot exclude an effect of unmeasured confounding. However, when adjusting for the most common clinical variables used to risk stratify patients with an MI, the 30‐day KCCQ scores carried independent prognostic significance. The registry enrolled consecutive patients with MI, but ≈34% of patients did not complete KCCQ within 30 days of discharge. While most clinical variables were similar in both groups, we cannot exclude an influence of a selection bias. Finally, as a single‐center study, validation of these findings is warranted to support their generalizability.

## Conclusion

PROs provide a practical and inexpensive means to identify HF symptoms. We show that remote evaluation of HF symptoms using the KCCQ score among patients recently discharged for MI identifies patients at risk for mortality. Whether closer follow‐up and targeted therapy can reduce mortality in these at‐risk patients warrants further investigation.

## Sources of Funding

This work was supported by the Ministry of Health of the Czech Republic (grant number NV 19–09‐00125, NU 22–02‐00130) and by the project National Institute for Research of Metabolic and Cardiovascular Diseases (Programme EXCELES, Project No. LX22NPO5104) funded by the European Union—Next Generation EU.

## Disclosures

Dr Wohlfahrt has received consulting fees or honoraria from Servier, outside the submitted work. Dr Kautzner reports grants and personal fees from Biosense Webster, Biotronik, Boston Scientific, and Medtronic; grants and personal fees from Abbott (SJM); and personal fees from Merit Medical, Daiichi Sankyo, Boehringer Ingelheim, BMS, Bayer, Merck, MSD, and Pfizer, all outside the submitted work. The remaining authors have no disclosures to report.

## Supporting information

Table S1
Figure S1

## References

[1] Wohlfahrt P , Stehlik J , Pan IZ , Ryan JJ . Empowering people living with heart failure. Heart Fail Clin. 2020;16:409–420. doi: 10.1016/j.hfc.2020.06.002 32888636

[2] Calkins DR , Rubenstein LV , Cleary PD , Davies AR , Jette AM , Fink A , Kosecoff J , Young RT , Brook RH , Delbanco TL . Failure of physicians to recognize functional disability in ambulatory patients. Ann Intern Med. 1991;114:451–454. doi: 10.7326/0003-4819-114-6-451 1825267

[3] Rumsfeld JS , Alexander KP , Goff DC , Graham MM , Ho PM , Masoudi FA , Moser DK , Roger VL , Slaughter MS , Smolderen KG , et al. Cardiovascular health: the importance of measuring patient‐reported health status. Circulation. 2013;127:2233–2249. doi: 10.1161/CIR.0b013e3182949a2e 23648778

[4] Heidenreich PA , Spertus JA , Jones PG , Weintraub WS , Rumsfeld JS , Rathore SS , Peterson ED , Masoudi FA , Krumholz HM , Havranek EP , et al. Health status identifies heart failure outpatients at risk for hospitalization or death. J Am Coll Cardiol. 2006;47:752–756. doi: 10.1016/j.jacc.2005.11.021 16487840

[5] Pokharel Y , Khariton Y , Tang Y , Nassif ME , Chan PS , Arnold SV , Jones PG , Spertus JA . Association of serial Kansas City Cardiomyopathy Questionnaire assessments with death and hospitalization in patients with heart failure with preserved and reduced ejection fraction: a secondary analysis of 2 randomized clinical trials. JAMA Cardiol. 2017;2:1315–1321. doi: 10.1001/jamacardio.2017.3983 29094152 PMC5814994

[6] Mishra RK , Yang W , Roy J , Anderson AH , Bansal N , Chen J , DeFilippi C , Delafontaine P , Feldman HI , Kallem R , et al. Kansas City Cardiomyopathy Questionnaire score is associated with incident heart failure hospitalization in patients with chronic kidney disease without previously diagnosed heart failure. Circ: Heart Fail. 2015;8:702–708. doi: 10.1161/CIRCHEARTFAILURE.115.002097 25985796 PMC4512877

[7] Hu D , Liu J , Zhang L , Bai X , Tian A , Huang X , Zhou K , Gao M , Ji R , Miao F , et al. Health status predicts short‐ and long‐term risk of composite clinical outcomes in acute heart failure. JACC: Heart Failure. 2021;9:861–873. doi: 10.1016/j.jchf.2021.06.015 34509406

[8] Parissis JT , Nikolaou M , Farmakis D , Paraskevaidis IA , Bistola V , Venetsanou K , Katsaras D , Filippatos G , Kremastinos DT . Self‐assessment of health status is associated with inflammatory activation and predicts long‐term outcomes in chronic heart failure. Eur J Heart Fail. 2009;11:163–169. doi: 10.1093/eurjhf/hfn032 19168514 PMC2639408

[9] Soto GE , Jones P , Weintraub WS , Krumholz HM , Spertus JA . Prognostic value of health status in patients with heart failure after acute myocardial infarction. Circulation. 2004;110:546–551. doi: 10.1161/01.CIR.0000136991.85540.A9 15262843

[10] Byrne RA , Rossello X , Coughlan JJ , Barbato E , Berry C , Chieffo A , Claeys MJ , Dan G‐A , Dweck MR , Galbraith M , et al. 2023 ESC Guidelines for the management of acute coronary syndromes: developed by the Task Force on the management of acute coronary syndromes of the European Society of Cardiology (ESC). Eur Heart J. 2023;44:3720–3826. doi: 10.1093/eurheartj/ehad191 37622654

[11] Aragam KG , Tamhane UU , Kline‐Rogers E , Li J , Fox KA , Goodman SG , Eagle KA , Gurm HS . Does simplicity compromise accuracy in ACS risk prediction? A retrospective analysis of the TIMI and GRACE risk scores. PLoS One. 2009;4:e7947. doi: 10.1371/journal.pone.0007947 19956773 PMC2776353

[12] D'Ascenzo F , Biondi‐Zoccai G , Moretti C , Bollati M , Omedè P , Sciuto F , Presutti DG , Modena MG , Gasparini M , Reed MJ , et al. TIMI, GRACE and alternative risk scores in acute coronary syndromes: a meta‐analysis of 40 derivation studies on 216,552 patients and of 42 validation studies on 31,625 patients. Contemp Clin Trials. 2012;33:507–514. doi: 10.1016/j.cct.2012.01.001 22265976

[13] Fox KA , Fitzgerald G , Puymirat E , Huang W , Carruthers K , Simon T , Coste P , Monsegu J , Gabriel Steg P , Danchin N , et al. Should patients with acute coronary disease be stratified for management according to their risk? Derivation, external validation and outcomes using the updated GRACE risk score. BMJ Open. 2014;4:e004425. doi: 10.1136/bmjopen-2013-004425 PMC3931985 24561498

[14] Jenča D , Melenovský V , Stehlik J , Staněk V , Kettner J , Kautzner J , Adámková V , Wohlfahrt P . Heart failure after myocardial infarction: incidence and predictors. ESC Heart Fail. 2021;8:222–237. doi: 10.1002/ehf2.13144 33319509 PMC7835562

[15] Gerber Y , Weston SA , Enriquez‐Sarano M , Berardi C , Chamberlain AM , Manemann SM , Jiang R , Dunlay SM , Roger VL . Mortality associated with heart failure after myocardial infarction: a contemporary community perspective. Circ Heart Fail. 2016;9:e002460. doi: 10.1161/CIRCHEARTFAILURE.115.002460 26699392 PMC4692179

[16] Wohlfahrt P , Jenča D , Melenovský V , Šramko M , Kotrč M , Želízko M , Mrázková J , Adámková V , Pitha J , Kautzner J . Trajectories and determinants of left ventricular ejection fraction after the first myocardial infarction in the current era of primary coronary interventions. Front Cardiovasc Med. 2022;9:1051995. doi: 10.3389/fcvm.2022.1051995 36451922 PMC9702523

[17] Thygesen K , Alpert JS , Jaffe AS , Chaitman BR , Bax JJ , Morrow DA , White HD ; Executive Group on behalf of the Joint European Society of Cardiology (ESC)/American College of Cardiology (ACC)/American Heart Association (AHA)/World Heart Federation (WHF) Task Force for the Universal Definition of Myocardial Infarction . Fourth universal definition of myocardial infarction (2018). Circulation. 2018;138:e618–e651. doi: 10.1161/CIR.0000000000000617 30571511

[18] Spertus JA , Jones PG , Sandhu AT , Arnold SV . Interpreting the Kansas City Cardiomyopathy Questionnaire in clinical trials and clinical care: JACC state‐of‐the‐art review. J Am Coll Cardiol. 2020;76:2379–2390. doi: 10.1016/j.jacc.2020.09.542 33183512

[19] Kosiborod M , Soto GE , Jones PG , Krumholz HM , Weintraub WS , Deedwania P , Spertus JA . Identifying heart failure patients at high risk for near‐term cardiovascular events with serial health status assessments. Circulation. 2007;115:1975–1981. doi: 10.1161/CIRCULATIONAHA.106.670901 17420346

[20] Dunlay SM , Gheorghiade M , Reid KJ , Allen LA , Chan PS , Hauptman PJ , Zannad F , Maggioni AP , Swedberg K , Konstam MA , et al. Critical elements of clinical follow‐up after hospital discharge for heart failure: insights from the EVEREST trial. Eur J Heart Fail. 2010;12:367–374. doi: 10.1093/eurjhf/hfq019 20197265 PMC3732083

[21] Wohlfahrt P , Jenča D , Stehlik J , Melenovský V , Mrázková J , Staněk V , Kettner J , Šramko M , Želízko M , Adámková V , et al. Heart failure‐related quality‐of‐life impairment after myocardial infarction. Clin Res Cardiol. 2023;112:39–48. doi: 10.1007/s00392-022-02008-z 35304902

[22] Ibanez B , James S , Agewall S , Antunes MJ , Bucciarelli‐Ducci C , Bueno H , Caforio ALP , Crea F , Goudevenos JA , Halvorsen S , et al. 2017 ESC Guidelines for the management of acute myocardial infarction in patients presenting with ST‐segment elevation: the Task Force for the management of acute myocardial infarction in patients presenting with ST‐segment elevation of the European Society of Cardiology (ESC). Eur Heart J. 2018;39:119–177. doi: 10.1093/eurheartj/ehx393 28886621

[23] Sulo G , Igland J , Vollset SE , Nygård O , Ebbing M , Sulo E , Egeland GM , Tell GS . Heart failure complicating acute myocardial infarction; burden and timing of occurrence: a nation‐wide analysis including 86 771 patients from the Cardiovascular Disease in Norway (CVDNOR) project. J Am Heart Assoc. 2016;5:e002667. doi: 10.1161/JAHA.115.002667 26744379 PMC4859383

[24] Fanaroff AC , Roe MT , Clare RM , Lokhnygina Y , Navar AM , Giugliano RP , Wiviott SD , Tershakovec AM , Braunwald E , Blazing MA . Competing risks of cardiovascular versus noncardiovascular death during long‐term follow‐up after acute coronary syndromes. J Am Heart Assoc. 2017;6:e005840. doi: 10.1161/JAHA.117.005840 28923989 PMC5634257

