# Supplementary material for: Remote Heart Failure Symptoms Assessment After Myocardial Infarction Identifies Patients at Risk for Death
Source: J Am Heart Assoc. 2024 Jan 9;13(2):e032505. doi: 10.1161/JAHA.123.032505 (PMC10926820; doi:10.1161/JAHA.123.032505)
Supplement: Supplementary file 1 — Table S1 Figure S1 [file JAH3-13-e032505-s001.pdf]

SUPPLEMENTAL MATERIAL

Table S1. Comparison of patients with missing and available KCCQ.

	KCCQ missing N=565	KCCQ available N=1135	P
Age	66.2±13.2	64.4±11.9	0.006
Sex (female)	177 (31.3)	303 (26.7)	0.052
BMI	28.0±5.2	28.8±4.9	0.001
CPR	38 (6.7)	34 (3.0)	0.001
Admission SBP	141±28	145±27	0.006
Admission DBP	78±15	79±13	0.125
Admission HR	81±20	76±18	0.0001
Maximal troponin, log	6.97±1.52	6.86±1.55	0.126
Creatinine	98.3±60.6	93.4±57.1	0.098
HbA1c	47.74±15.00	45.21±12.96	0.001
STEMI	320 (56.6)	682 (60.1)	0.174
Killip class I	383 (67.8)	931 (82.0)	0.001
EF	43±11	46±10	0.001
EF below 40%	155 (27.4)	254 (22.4)	0.022
Death, n(%)	84 (14.9)	146 (12.9)	0.260

Figure S1. Calibration plot for model based on clinical variables (A) and clinical variables with addition of three KCCQ categories (B).

A

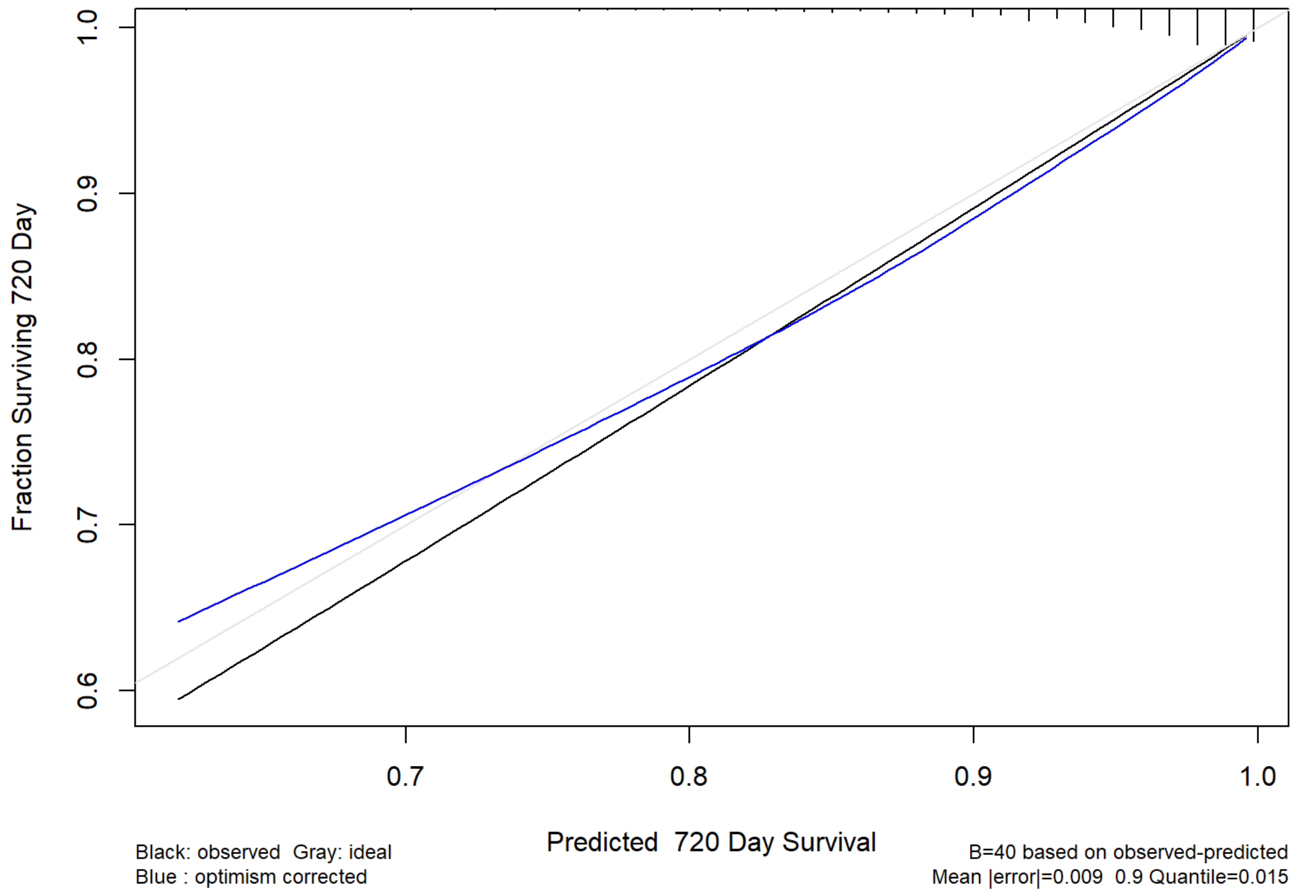

B

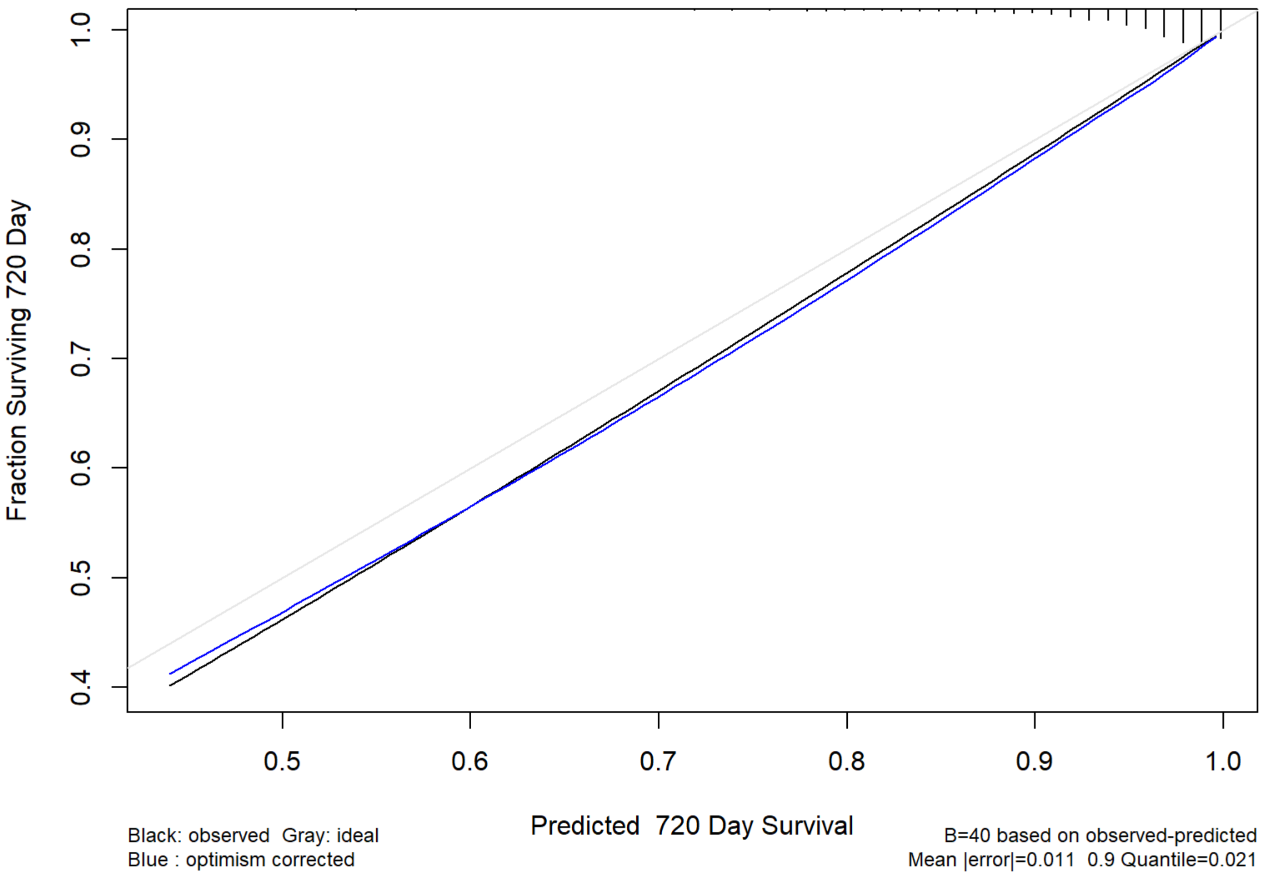
Fraction Surviving 720 Day
0.4
0.5
0.6
0.7
0.8
0.9
1.0
0.5
0.6
0.7
0.8
0.9
1.0
Predicted 720 Day Survival
Black: observed Gray: ideal
Blue : optimism corrected
B=40 based on observed-predicted
Mean |error|=0.011 0.9 Quantile=0.021
